# Progress in Gynæcology

**Published:** 1897-04-17

**Authors:** 


### Progress in Gynæcology.

**Gonorrhœa in Women.**—Statistics as to the frequency of this disease compiled by Noeggerath and Oppenheimer are quoted by Bernard Gordon[1]; the former states that he found evidence of gonorrhœa in 80 per cent. of the woman examined by him, the latter found that out of 108 pregnant women examined by him 27·7 per cent. had gonorrhœa. Gordon found that gonorrhœal affection of the tubes was less frequent than that of the uterus, and was characterised by thickening of mucous membrane, destruction of ciliated epithelium, in some cases thickening of the underlying connective tissue, and infiltration and thickening of the tube wall, with atrenia and stenosis of the tube, and frequent attacks of peritonitis, the affection usually terminating in sterility. The arguments for gonorrhœa being a specific inflammation due to a specal micro-organism, the gonococcus of Neisser, are given by George Newton,[2] instead of a simple inflammation due to staphylococci and others which are often found to be present with the gonococcus to complicate its effects, and to which are attributed the joint and endocardial troubles met with in this disease. In an abstract of a discussion on this subject taken from the *Centralblatt für Gynakologie* W. J. Sinclair[3] lays stress equally with Newton on the importance of microscopical examination not only in all suspected cases but in those where infection is certain; in the suspected cases the doubt will be removed, in the known ones the cessation of infectivity can be determined. The discharge examined should be taken from several localities, from (1) the urethra, (2)

the folds about the urethral orifice, (3) the cervical canal, and (4) the orifices of the glands of Bartholin. The examination is of the more importance since clinical symptoms may be absent, although the gonococcus is present, and acute inflammation may be caused with the symptoms of gonorrhœa without the presence of the gonococcus. The differentiation of the case is very important; the non-specific inflammation runs a rapid course and leaves scarcely any serious results, the specific inflammation may be most obstinate, and lead to permanent injury of the pelvic organs. Hence the necessity for proving the specific nature of the disease, and treating it as such by means of remedies which, while not irritating the mucous membrane, act as germicides to the gonococcus, and do not lose their germicide effect if combined with albumin or mucin. That many chronic diseases of the pelvic organs and genital tract, originally due to gonorrhœal infection, do not continue to depend on the presence and activity of the gonococcus, whose life is short, but upon tissue changes originally set up by it, which still continue and develop further, is shown by Neisser.[4] Among these are the changes in the pelvic peritoneum, ovaries, and Fallopian tubes, endometritis, colpitis and urethritis maculosa, and colpitis granulosa, adenitis and cystic disease of Bartholin's glands, and vulvitis maculosa. Vulvitis urethritis, and colpitis maculosa he describes as patches of dark red colouration of these parts, varying from punctiform spots to patches the size of a horse-bean.

*Treatment* should first of all be directed to the relief of the more pressing symptoms, *e.g.*, dysuria, smarting pain, and irritation, by the use of diluents, alkalis, and local sedatives; but special local treatment should be directed to the destruction of the micro-organism. In addition to nitrate of silver solution, perchloride of mercury solution, and permanganate of potash, Gordon[5] recommends solutions of argentamine 1-4,000, and argonine 1 or 1½ per cent. Sinclair,[6] in addition to the above, advises the use of oxycyanate of mercury and icthyol, which latter has in his hands given excellent results, solutions of a strength of 1-200 causing the disappearance of the micro-organism in a few days.

[1] American Med. Surg. Bulletin, Nov. 14, 1896. [2] Chicago Med. Review, January, 1897. [3] Med. Chron., Dec., 1896. [4] Med. Chron. loc. cit. [5] Amer. Med. Surg. Bulletin, Nov. 14, 1896, loc. cit. [6] Med. Chron., loc. cit.

